# Synthesis and Characterization of PU/PLCL/CMCS Electrospun Scaffolds for Skin Tissue Engineering

**DOI:** 10.3390/polym14225029

**Published:** 2022-11-19

**Authors:** Xiang Gao, Meiling Wen, Yang Liu, Tian Hou, Bin Niu, Meiwen An

**Affiliations:** College of Biomedical Engineering, Taiyuan University of Technology, Taiyuan 030024, China

**Keywords:** polyurethane, poly(L-lactide-co-caprolactone), carboxymethyl chitosan, electrospun fiber, cytocompatibility, skin tissue engineering

## Abstract

As tissue regeneration material, electrospun fibers can mimic the microscale and nanoscale structure of the natural extracellular matrix (ECM), which provides a basis for cell growth and achieves organic integration with surrounding tissues. At present, the challenge for researchers is to develop a bionic scaffold for the regeneration of the wound area. In this paper, polyurethane (PU) is a working basis for the subsequent construction of tissue-engineered skin. poly(L-lactide-co-caprolactone) (PLCL)/carboxymethyl chitosan (CMCS) composite fibers were prepared via electrospinning and cross-linked by glutaraldehyde. The effect of CMCS content on the surface morphology, mechanical properties, hydrophilicity, swelling degree, and cytocompatibility were explored, aiming to assess the possibility of composite scaffolds for tissue engineering applications. The results showed that randomly arranged electrospun fibers presented a smooth surface. All scaffolds exhibited sufficient tensile strength (5.30–5.60 MPa), Young’s modulus (2.62–4.29 MPa), and swelling degree for wound treatment. The addition of CMCS improved the hydrophilicity and cytocompatibility of the scaffolds.

## 1. Introduction

The skin is the largest organ of the human body and the first barrier against outside pathogens. It is highly susceptible to external mechanical, chemical, and pathogenic microbial attacks, resulting in chronic wounds [1,2]. Conventional treatments, such as allogeneic or autologous skin grafts, are limited by immune rejection and insufficient donors, preventing their wide application [3]. Skin tissue engineering scaffolds have a great demand in the treatment of full-thickness wounds. Therefore, the development of high-performance tissue-engineered scaffolds is of great importance for the treatment of patients with skin defects.

At present, methods for preparing tissue-engineered scaffolds include 3D printing [4], 4D printing [5], et al. Among these many methods, electrospinning is a sophisticated preparation method that is widely used in the field of tissue engineering. Electrospinning is a technique for the production of microscale and nanoscale polymer fibers. By using different types of synthetic or natural polymers to prepare scaffold materials, it is possible to meet the requirements of different tissues in terms of mechanical properties and degradation properties. At the same time, the diameter and topology of electrospun fibers can be flexibly adjusted, which is more conducive to mimicking the structure of the natural extracellular matrix (ECM). In this case, cell proliferation and tissue regeneration become feasible [6,7,8,9,10]. Hybrid electrospun fibers of natural and synthetic polymers can be fabricated into an excellent scaffold with good physicochemical properties and biocompatibility [11,12,13]. Yang et al. found that electrospun fibers of poly(lactic-co-glycolic acid (PLGA) mixed with collagen enhanced cell attachment and proliferation [14]. Chong et al. discovered the great potential of electrospun polycaprolactone (PCL)/gelatin nanofibrous for wound healing and layered dermal reconstruction [15].

Polyurethane (PU) is a semi-crystalline polymer with a combination of hard and soft sections, which not only supply attachment sites for human skin fibroblasts (HSFs) but also significantly reduce the proliferative scar contraction and scar stiffness caused by scaffold degradation [16,17,18]. PCL has a functional group similar to PU [19], which has been proven to be an ideal reinforcer and toughener for PU electrospun membranes. Poly(L-lactide-co-caprolactone) (PLCL) is formed by the random copolymerization of lactic acid and caprolactone, which has a comparable structure with PCL and good cytocompatibility [20,21,22]. Moreover, PLCL can be co-blended with a variety of materials for electrospinning.

Carboxymethyl chitosan (CMCS) is a water-soluble derivative of chitosan with functionalized chemical groups (-NH_2_ and -COOH), antioxidant, and antibacterial properties that promote wound healing and facilitate collagen secretion [23]. CMCS contains a large number of hydrophilic groups, which makes it extremely soluble in water, and glutaraldehyde cross-linking is often used to reduce solubility. However, the high brittleness of pure CMCS and harsh electrospinning conditions hinder its application in the field of biomedical materials. The combination of these three substances overcomes the deficiencies of the individual substances and improves the properties of the material, which has a greater prospect of application.

In this paper, electrospun fibers with different CMCS contents were prepared by co-blending electrospinning. Electrospun PU/PLCL/CMCS fibrous scaffolds have microscale structures and large specific areas, which can simulate the function and structure of the natural ECM. The surface morphology, mechanical properties, hydrophilicity, and cytocompatibility were characterized to demonstrate that the prepared novel biomaterials have adequate mechanical strength and good cytocompatibility. We expect that the composite scaffolds can meet the complex requirements of cell and new skin tissue growth, and provide some research basis for subsequent application as skin tissue engineering scaffolds for wound repair.

## 2. Materials and Methods

### 2.1. Materials

PU was supplied by Sigma-Aldrich Sigma Trading Co., Ltd., Beijing, China. PLCL (LA/CL = 70/30, 200 kDa) was bought from Yongkang Leye. CMCS (Carboxylation degree: 87–90%) was provided by Solarbio. Chloroform (TCM) was purchased from Shentai Chemical Reagent Co., Ltd., Tianjin, China. N, N-dimethylformamide (DMF) was provided by Tianjin Huihang Chemical Technology Co., Ltd., Tianjin, China. All chemicals and solvents were of reagent grade.

### 2.2. Preparation of PU/PLCL/CMCS Blended Solution

The best proportion of PU and PLCL co-blended electrospinning solution was determined by the previous study of the group [24]. A certain mass of PU and PLCL was weighed and dissolved in the mixed solution of DMF and TCM (1/1, *v*/*v*), and stirred with a magnetic stirrer at 30° C for 15 h. After the solution was mixed well, different proportions of CMCS were added to obtain polymer solutions with different compositions, as described in Table 1.

### 2.3. Fabrication of Electrospun Membranes

**Figure 1:**
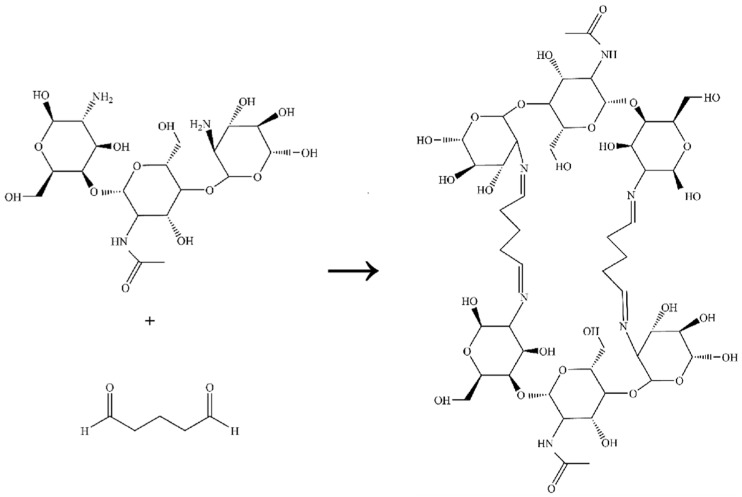
Principle of the glutaraldehyde cross-linking reaction.

Electrospinning was performed using a high-voltage electrospinning machine (Tianjin Yunfan Technology Co., Tianjin, China). A flat-tipped stainless needle (20 gauge, ID = 0.6 mm, OD = 0.9 mm) was fixed on a 5 mL disposable syringe containing polymer solution for the experiments. The electrospinning process of composite scaffolds was controlled at a flow rate of 1–1.2 mL/h, a high voltage of 15 kV, and a collected distance of 15–20 cm. To promote solvent evaporation and fiber stretching, the ambient temperature was controlled at 30–35 °C and the humidity was kept at 25–30%. In order to study the effect of CMCS, all the electrospinning parameters and experimental conditions were kept constant. The obtained electrospun membranes were dried in a vacuum drying oven for more than 96 h to remove the residual solvent. After that, the electrospun membranes were cross-linked by 10% glutaraldehyde steam at room temperature for 3 h [25,26]. Then the materials were removed and immersed in glycine solution for 30 min to eliminate the remaining glutaraldehyde. The principle of glutaraldehyde crosslinking reaction is shown in Figure 1.

### 2.4. Surface Morphology and Chemical Structure

Scanning electron microscopy (SEM, JSM-7100F, Tokyo, Japan) was used to view the morphological structure of electrospun membranes. The membranes were cut into small pieces of 0.5 cm × 0.5 cm and glued to the black conductive adhesive before detection. After gold coating, the sample was observed and photographed at an operating voltage of 10 kV [27,28]. An infrared spectral diffraction analyzer (Bruker Alpha, Karlsruhe, Germany) was utilized to detect the type of chemical bonds before and after cross-linking on the surface of electrospun membranes. The total reflection infrared test was performed on the electrospun membranes and CMCS powder after vacuum drying treatment, and the measured wavelength range was 400 cm^−1^–4000 cm^−1^ with a resolution of 4 cm^−1^ [29].

### 2.5. Swelling Properties

Dry electrospun membranes (about 20 mg) were completely immersed in distilled water overnight. After gently wiping off the surface liquid with absorbent paper, the samples were accurately weighed [30]. The swelling rate was calculated using the following formula [31]:

$$S_{W}=\frac{W_{2}-W_{1}}{W_{1}}\times 100\%$$

W_2_ and W_1_ refer to the weight of the wet electrospun membranes and dry electrospun membranes, respectively.

### 2.6. Hydrophilicity

To evaluate the hydrophilicity of electrospun membranes, the water contact angle (WCA) on the samples was measured using an optical contact angle goniometer (Optima, Beijing Wuzhou Oriental Technology Development Co., Ltd., Beijing, China). The membranes were cut into small pieces of 1 cm × 1 cm. Using 2 µL of deionized water as the test liquid, the WCA was recorded at 120 s and averaged six times for each sample tested [29,32,33].

### 2.7. Mechanical Properties

The mechanical properties of electrospun membranes were measured using a universal mechanical testing machine (Instron 5544, Boston, MA, USA). The materials were cut into rectangular strips with a width of 8 mm and a length of 60 mm and were mounted vertically on the tester’s gripping device. Since thickness is a key factor affecting the mechanical results, the sample thickness was tested by applying a laser displacement measurement sensor (LK-G5000, Ōsaka, JPN). Using a 50 N force measuring transducer, the materials were pulled at a rate of 5 mm/min [19]. Tensile strength and ultimate elongation at break were calculated from the stress-strain curve, and Young’s modulus was obtained in the elastic region of the curve.

### 2.8. Cell Compatibility

The viability and proliferation of HSFs on scaffolds were monitored using the Cell Counting Kit-8 (CCK-8). The cells were obtained from Shanxi Bethune Hospital. Electrospun membranes were made into 10 mm diameter discs. Triple parallel samples of each group were placed on the bottom of 48-well plates. After sterilization of the samples with ultraviolet (200–280 nm) for 1 h, HSFs were cultured onto different electrospun membranes at a density of 1 × 10^4^ cells/well, 300 μL of complete medium was added, and the medium was changed every two days. The proliferation rate of HSFs was determined by CCK-8 at 1, 4, and 7 day(s), respectively [27,34]. The light absorbance was computed at 450 nm using a microplate reader (Biorad iMark, Hercules, CA, USA).

Phalloidin-iFluor 488 was used to detect the spreading skeletal morphology of the HSFs. After cells were incubated on electrospun membranes for 1, 4, and 7 day(s), cells were washed with PBS and fixed in 4% paraformaldehyde for 30 min, followed by three washes with PBS. Triton X-100 (0.1%) was used to increase permeability. Finally, cells were incubated with 50 μL of diluted 1 × phalloidin for 60 min in the dark, and the nuclei were labeled with DAPI [32,34]. Fluorescence images were acquired using a confocal scanning microscope (Leica, Wetzlar, Germany).

The adhesion and spreading of HSFs on the scaffolds were analyzed by SEM. HSFs were seeded onto the electrospun membrane and incubated for 1 and 3 day(s). After being fixed with 4% paraformaldehyde, the cell-scaffold structure was washed with PBS three times. The samples were dehydrated with ethanol solution at gradient concentrations (30%, 40%, 50%, 60%, 70%, 80%, 90%, 95%, and 100%). The dried samples were sprayed with gold and observed by SEM [27].

### 2.9. Statistical Analysis

The resulting data were expressed as mean ± standard deviation of at least five measurements. All data were evaluated by Student’s *t*-test (single comparisons) or one-way ANOVA test (multiple comparisons) as * *p* < 0.05, ** *p* < 0.01, *** *p* < 0.001.

## 3. Results and Discussion

### 3.1. Morphology of Fibrous Scaffolds

**Figure 2:**
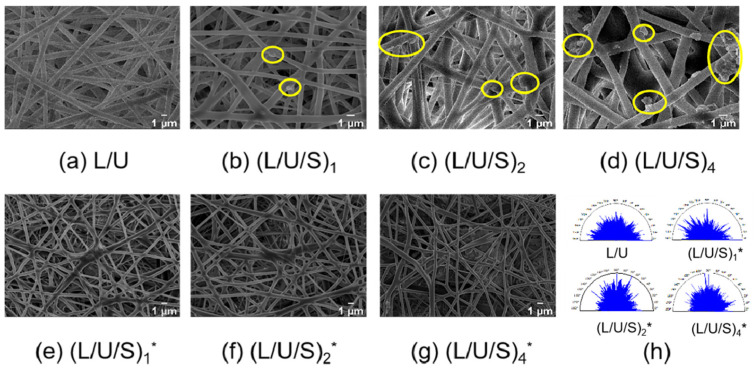
(**a**–**g**) SEM images of electrospun fibers with different CMCS content before and after cross-linking. (**h**) Polar coordinates of fiber texture direction. CMCS particles are circled by yellow solid line ellipses. (L/U/S)_1_^*^, (L/U/S)_2_^*^, (L/U/S)_4_^*^ referred to the sample after (L/U/S)_1_, (L/U/S)_2_, (L/U/S)_4_ were cross-linked, respectively.

**Figure 3:**
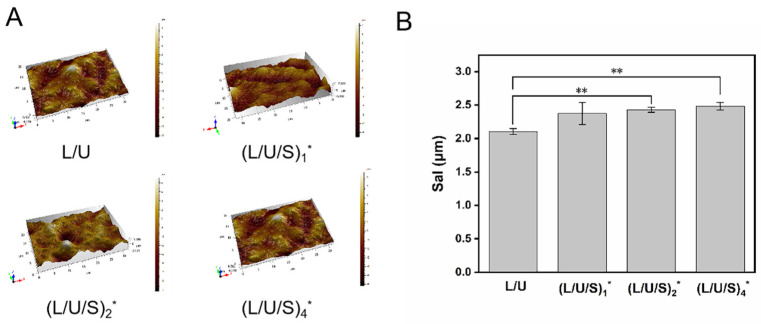
(**A**) Three-dimensional surface height pattern diagram of electrospun fibers, (**B**) analysis of smoothness parameters of electrospun fibers. (L/U/S)_1_^*^, (L/U/S)_2_^*^, (L/U/S)_4_^*^ referred to the sample after (L/U/S)_1_, (L/U/S)_2_, (L/U/S)_4_ were cross-linked, respectively. ** *p* < 0.01.

In this work, PU/PLCL/CMCS fibrous scaffolds were fabricated by electrospinning. Figure S1 demonstrated the visual images of the electrospun membranes. The SEM images of the electrospun fibers before and after cross-linking are shown in Figure 2. It can be seen that before cross-linking, all electrospun fibers showed smooth and randomly arranged structures (Figure 2h) without beads and fractures. CMCS existed in the form of small particles on the fibers. The fiber diameters of all samples were similar. After cross-linking, the fibers become curved, and bonding between the fibers becomes obvious. The CMCS particles entered the interior of the fibers and were closely associated with fibers. The surface of electrospun fibers showed a rough morphology that is convenient for cell adhesion and spreading, which is also demonstrated in Figure 3a,b. Figure 3a showed the height pattern of the fiber surface, and Figure 3b assessed the surface roughness of the material by the Sal parameter, the smaller the value, the more smoothed the features. It has been shown that with the addition of CMCS, the Sal value of electrospun fibers increased, and the smoothness decreased. Moreover, the (L/U/S)_4_ electrospun solution more easily blocked the needle than the other groups, which also indicated that further increasing CMCS content will make the electrospinning process unfeasible.

### 3.2. Structure of Fibrous Scaffolds before and after Cross-Linking

**Figure 4:**
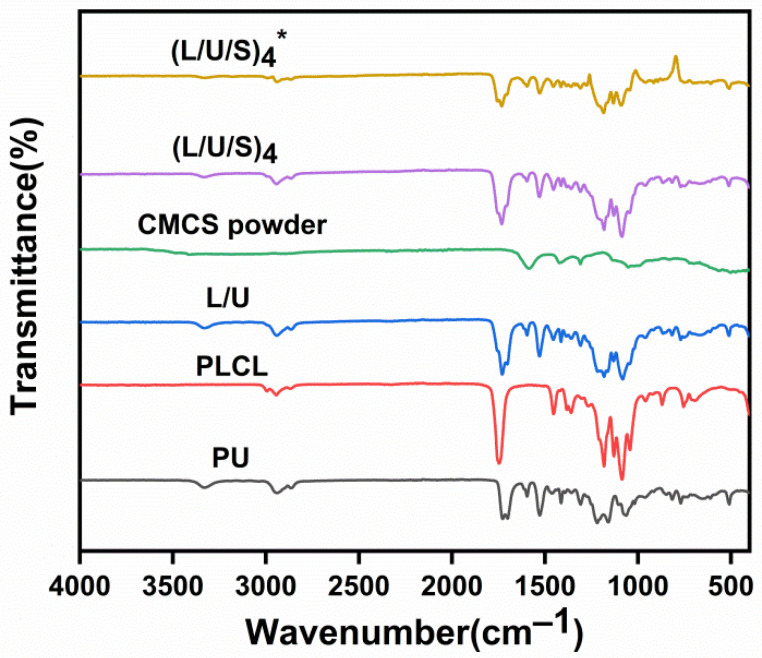
FTIR spectra of PU/PLCL/CMCS electrospun fibers. (L/U/S)_4_^*^ referred to the sample after (L/U/S)_4_ was cross-linked.

The chemical structure of the electrospun membranes’ surface was characterized by a Fourier transform infrared (FTIR) spectroscopy experiment. As shown in Figure 4, the stretching vibration peak of the C-N group was observed at 1309 cm^−^^1^ attributed to CMCS. Asymmetric and symmetric stretching vibration peaks of -COO- can be detected at 1585 cm^−^^1^. The major peaks at 1700 cm^−^^1^ and 1728 cm^−^^1^ were the stretching vibration peaks of C=O and C=N. The broad peak of N-H symmetric vibration at 3330 cm^−^^1^ proved the presence of PU. The peak at 1181 cm^−^^1^ represented the existence of C-O stretching vibrations in PLCL. The result showed that no new peak was formed by the addition of CMCS, but the shape of the peak at 1728 cm^−^^1^ broadened due to the formation of hydrogen bonding, electron cloud density averaging, and the frequency of the group stretching vibration was reduced. After cross-linking, the intensity of the peaks located at 3330 cm^−^^1^ and 1728 cm^−^^1^ was significantly weakened, indicating that the amino group in the electrospun membranes reacted chemically with glutaraldehyde to form a reticulated polymer [31,35,36].

### 3.3. Hydrophilicity of Fibrous Scaffolds

**Figure 5:**
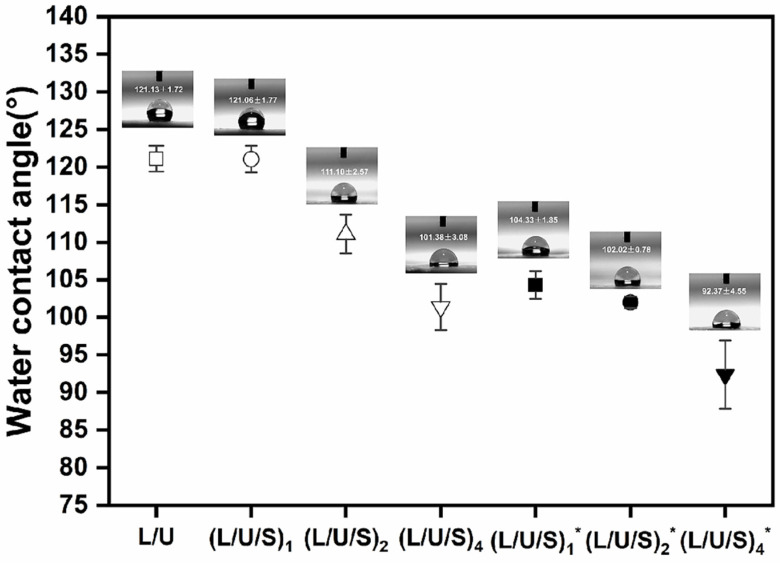
Water contact angle of PU/PLCL/CMCS fibers. (L/U/S)_1_^*^, (L/U/S)_2_^*^, (L/U/S)_4_^*^ referred to the sample after (L/U/S)_1_, (L/U/S)_2_, (L/U/S)_4_ were cross-linked, respectively.

It has been demonstrated that cell adhesion and growth on biomaterials were strongly dependent on surface hydrophilicity [27]. The smaller the water contact angle of the material surface, the more favorable cells’ adhesion and spreading. The surface hydrophilicity of electrospun membranes was influenced by the surface morphology and elemental composition [37]. As shown in Figure 5, the water contact angle decreased with the increase in CMCS content, indicating that the addition of CMCS improved the hydrophilicity of the material. This may be attributed to the abundant hydrophilic groups in CMCS, such as amino and hydroxyl groups [23]. It can quickly absorb water droplets on the surface of the material and promote cell adhesion and proliferation.

### 3.4. Mechanical Properties of Fibrous Scaffolds

**Figure 6:**
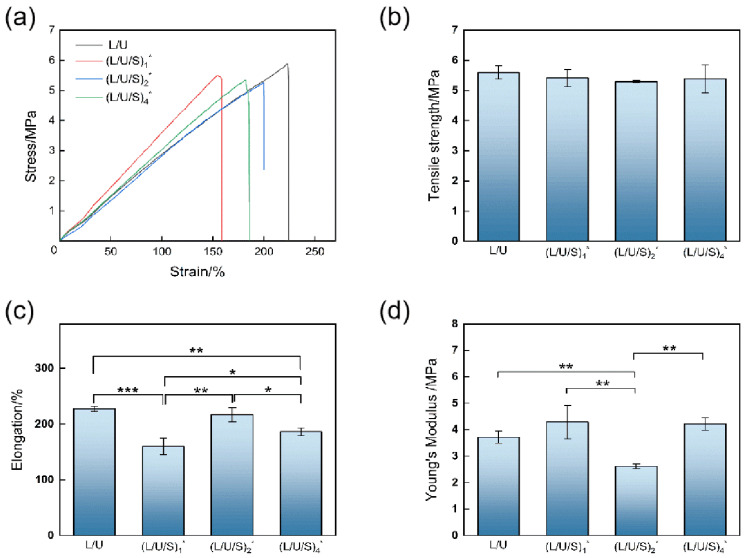
Mechnical Properties of different electrospun membranes: (**a**) Stress-strain curves; (**b**) Tensile strength; (**c**) Elongation; (**d**) Young’s modulus. (L/U/S)_1_^*^, (L/U/S)_2_^*^, (L/U/S)_4_^*^ referred to the sample after (L/U/S)_1_, (L/U/S)_2_, (L/U/S)_4_ were cross-linked, respectively. * *p* < 0.05, ** *p* < 0.01, *** *p* < 0.001.

The mechanical properties of the prepared electrospun membranes were measured by uniaxial tensile experiments, including Young’s modulus, tensile strength, and elongation at break. The sample dimensions before stretching and the images of the sample during stretching have been shown in Figures S2 and S3. On the stress-strain curves, all samples showed a short linear region with a large slope at the initial stage, followed by a longer linear region until pulling off. During the stretching phase, the electrospun fibers parallel to the stretching direction were the first to bear the stress. As the stretching process prolonged, the strain increased and the fibers in other directions begin to share the stress under tension, resulting in an improvement in the tensile strength of the electrospun membranes [38]. As shown in Figure 6, the tensile strength of electrospun fibers ranged from 5.30 ± 0.03 MPa to 5.60 ± 0.22 MPa, and Young’s modulus increased from 2.62 ± 0.09 MPa to 4.29 ± 0.62 MPa. Meanwhile, the composites have high elongation, and the maximum elongation can reach 227.77 ± 4.67%, indicating that PU/PLCL/CMCS had good flexibility. The results showed that the addition of CMCS had a small effect on the mechanical properties of the composite electrospun membranes. The prepared electrospun fibrous scaffolds were compatible with the tensile strength (5–40 MPa) and Young’s modulus (2.4–25 MPa) of human skin tissue engineering scaffolds reported in the literature [39].

The ideal tissue-engineered scaffolds are required to have an appropriate swelling rate in order to maintain a certain level of moisture in the wound area to promote wound healing. A swelling degree from 100% to 900% is a desirable range [40]. Table 2 showed the absorbent swelling degree of different electrospun membranes in PBS buffer. The swelling rate of all electrospun membranes was higher than 100%. After the addition of CMCS, the swelling rate of the fiber membranes presented a tendency to increase, probably due to the many hydrophilic groups of CMCS. During the cross-linking process, the amino groups in the CMCS molecular chains change from the bound state of intermolecular hydrogen bonds to the free state, resulting in an increased ability to absorb liquid [41]. In conclusion, the prepared composite scaffolds meet the requirements of tissue engineering scaffolds for the swelling degree.

### 3.5. Cytocompatibility of Fibrous Scaffolds

**Figure 7:**
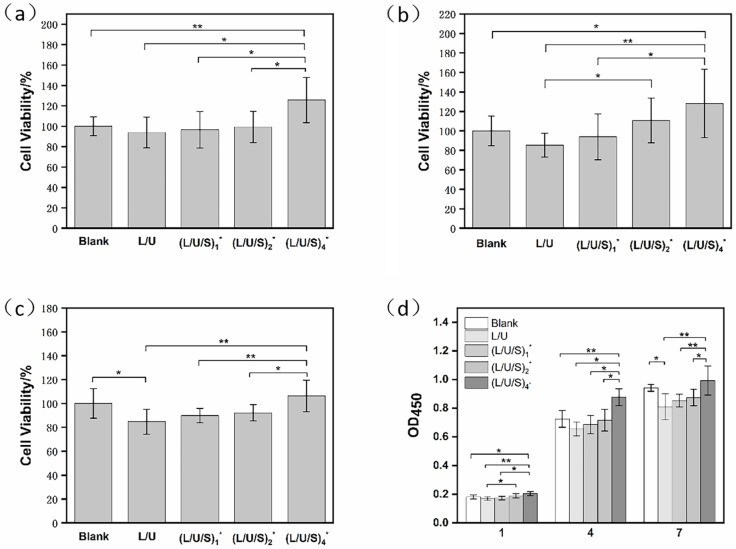
CCK-8 determined cell viability in four groups of electrospun membranes at 1 day (**a**), 4 days (**b**), and 7 days (**c**), and cell proliferation response OD value (**d**). (L/U/S)_1_^*^, (L/U/S)_2_^*^, (L/U/S)_4_^*^ referred to the sample after (L/U/S)_1_, (L/U/S)_2_, (L/U/S)_4_ were cross-linked, respectively. * *p* < 0.05, ** *p* < 0.01.

The purpose of this study was to fabricate composite scaffolds for application in skin tissue engineering. Therefore, PU/PLCL/CMCS composite scaffolds should have good cytocompatibility. The proliferation of HSFs on scaffolds was assessed using the CCK-8. The absorbance was measured at 450 nm using a microplate reader. As shown in Figure 7, the absorbance of the PU/PLCL/CMCS composite scaffold increased with the prolonged incubation time, which corresponds to the increase in HSFs. Moreover, the absorbance also showed an increasing tendency as the CMCS content increased, which suggested that the addition of CMCS promoted cell proliferation.

**Figure 8:**
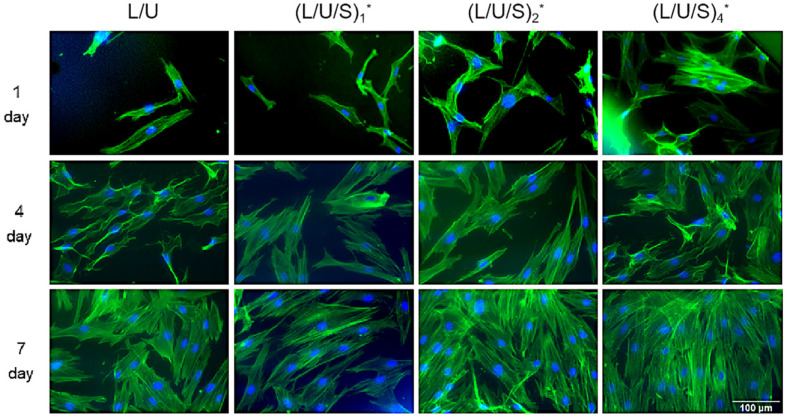
The cytoskeleton of HSFs on different electrospun membranes at 1, 4, and 7 day(s) (cytoskeleton-green fluorescence, nucleus-blue fluorescence). (L/U/S)_1_^*^, (L/U/S)_2_^*^, (L/U/S)_4_^*^ referred to the sample after (L/U/S)_1_, (L/U/S)_2_, (L/U/S)_4_ were cross-linked, respectively.

**Figure 9:**
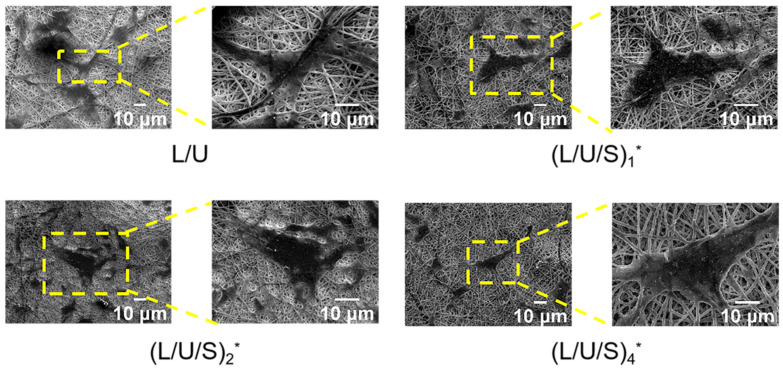
SEM images about adherence and morphology of HSFs after 48 h. (L/U/S)_1_^*^, (L/U/S)_2_^*^, (L/U/S)_4_^*^ referred to the sample after (L/U/S)_1_, (L/U/S)_2_, (L/U/S)_4_ were cross-linked, respectively.

The morphology of cells on electrospun fibers was studied after immunofluorescence staining, and as seen in Figure 8, the nucleus was labeled with blue fluorescence and the cytoskeleton was labeled with green fluorescence. The cells were extensively distributed and exhibited good intercellular interactions at 4 days, which facilitated the maintenance of cell viability and function. When the culture time reached 7 days, the cells existed as cell clusters. With the greater CMCS content, the cells exhibited a larger spreading area and more obvious stress fibers. This resulted in the whole field of view being almost occupied by cells (Figure 8). Figure 9 showed the morphology of HSFs cultured on the composite fiber scaffold for 48 h. The cells spread on the surface of the electrospun fiber scaffold, displaying a flat morphology and attaching to the fiber by extending pseudopodium. This result indicated that the composite fiber scaffold can provide a good cellular environment for the adhesion and proliferation of HSFs.

## 4. Conclusions

In this paper, PU/PLCL/CMCS composite scaffolds with different CMCS content were successfully prepared by electrospinning. The combination of CMCS with PU, and PLCL overcame the inherent brittleness of CMCS while compensating for the weak biocompatibility of synthetic polymers. After cross-linking by glutaraldehyde, CMCS was uniformly distributed on the surface and inside of the scaffolds. The tensile tests demonstrated that the composite scaffolds had high mechanical strength. The CCK-8 and immunofluorescence staining showed that the composite scaffold had good cytocompatibility and the ability to promote the attachment and proliferation of HSFs. These obtained results suggested that the composite PU/PLCL/CMCS scaffolds with natural ECM-like structures met the complex requirements for cellular and nascent skin tissue growth, and might be a potential biomaterial that can be applied to skin tissue engineering.

## Figures and Tables

**Table 1:** Compositions of the polymer solutions for electrospun membranes.

Sample	Composite Weight Ratios (PU/PLCL/CMCS)	PU (mg/mL)	PLCL (mg/mL)	CMCS (mg/mL)
L/U	4:4:0	65	65	0
(L/U/S)_1_	4:4:1	65	65	16.25
(L/U/S)_2_	4:4:2	65	65	32.5
(L/U/S)_4_	4:4:4	65	65	65

PU-Polyurethane; PLCL-Poly(L-Lactide-co-caprolactone); CMCS-Carboxymethyl chitosan.

**Table 2:** Swelling degree of PU/PLCL/CMCS electrospun fibers.

Sample	Swelling Degree/%
L/U (L/U/S)_1_^*^ (L/U/S)_2_^*^	117 ± 13 201 ± 15 265 ± 47
(L/U/S)_4_^*^	285 ± 3

(L/U/S)_1_*, (L/U/S)_2_*, (L/U/S)_4_* referred to the sample after (L/U/S)_1_, (L/U/S)_2_, (L/U/S)_4_ were cross-linked, respectively.

## Data Availability

The data generated from the study is clearly presented and discussed in the manuscript.

## Supplementary Materials

The following supporting information can be downloaded at: https://www.mdpi.com/article/10.3390/polym14225029/s1, Figure S1: Visual images of the electrospun fiber membrane prepared with different CMCS content; Figure S2: Original dimension of the electrospun membrane sample before stretching; Figure S3: Images of the electrospun membrane sample in the stretching process; Figure S4: Image of cell culture plated in the methods CCK-8.

## References

[1] Xiong S., Zhang X., Lu P., Wu Y., Wang Q., Sun H., Heng B.C., Bunpetch V., Zhang S., Ouyang H.A. (2017). Gelatin-sulfonated Silk Composite Scaffold based on 3D Printing Technology Enhances Skin Regeneration by Stimulating Epidermal Growth and Dermal Neovascularization. Sci. Rep..

[2] Olsson M., Jarbrink K., Divakar U., Bajpai R., Upton Z., Schmidtchen A., Car J. (2019). The humanistic and economic burden of chronic wounds: A systematic review. Wound Repair Regen..

[3] Nosrati H., Aramideh Khouy R., Nosrati A., Khodaei M., Banitalebi-Dehkordi M., Ashrafi-Dehkordi K., Sanami S., Alizadeh Z. (2021). Nanocomposite scaffolds for accelerating chronic wound healing by enhancing angiogenesis. J. Nanobiotechnol..

[4] Soleyman E., Aberoumand M., Rahmatabadi D., Soltanmohammadi K., Ghasemi I., Baniassadi M., Abrinia K., Baghani M. (2022). Assessment of controllable shape transformation, potential applications, and tensile shape memory properties of 3D printed PETG. J. Mater. Res. Technol..

[5] Soleyman E., Rahmatabadi D., Soltanmohammadi K., Aberoumand M., Ghasemi I., Abrinia K., Baniassadi M., Wang K., Baghani M. (2022). Shape memory performance of PETG 4D printed parts under compression in cold, warm, and hot programming. Smart Mater. Struct..

[6] Xue J.J., Wu T., Dai Y.Q., Xia Y.N. (2019). Electrospinning and Electrospun Nanofibers: Methods, Materials, and Applications. Chem. Rev..

[7] Barhoum A., Pal K., Rahier H., Uludaǧ H., Kim I.S., Bechelany M. (2019). Nanofibers as new-generation materials: From spinning and nano-spinning fabrication techniques to emerging applications. Appl. Mater. Today.

[8] Sabra S., Ragab D.M., Agwa M.M., Rohani S. (2020). Recent advances in electrospun nanofibers for some biomedical applications. Eur. J. Pharm. Sci..

[9] Miguel S.P., Ribeiro M.P., Coutinho P., Correia I.J. (2017). Electrospun Polycaprolactone/Aloe Vera_Chitosan Nanofibrous Asymmetric Membranes Aimed for Wound Healing Applications. Polymers.

[10] Cui W., Zhou Y., Chang J. (2010). Electrospun nanofibrous materials for tissue engineering and drug delivery. Sci. Technol. Adv. Mat..

[11] Sundaramurthi D., Krishnan U.M., Sethuraman S. (2014). Electrospun Nanofibers as Scaffolds for Skin Tissue Engineering. Polym. Rev..

[12] Parham S., Kharazi A.Z., Bakhsheshi-Rad H.R., Ghayour H., Ismail A.F., Nur H., Berto F. (2020). Electrospun Nano-Fibers for Biomedical and Tissue Engineering Applications: A Comprehensive Review. Materials.

[13] Ye K., Kuang H., You Z., Morsi Y., Mo X. (2019). Electrospun Nanofibers for Tissue Engineering with Drug Loading and Release. Pharmaceutics.

[14] Yang Y., Zhu X., Cui W., Li X., Jin Y. (2009). Electrospun Composite Mats of Poly [(D,L-lactide)-co-glycolide] and Collagen with High Porosity as Potential Scaffolds for Skin Tissue Engineering. Macromol. Mater. Eng..

[15] Chong E.J., Phan T.T., Lim I.J., Zhang Y.Z., Bay B.H., Ramakrishna S., Lim C.T. (2007). Evaluation of electrospun PCL/gelatin nanofibrous scaffold for wound healing and layered dermal reconstitution. Acta Biomater..

[16] Lorden E.R., Miller K.J., Ibrahim M.M., Bashirov L., Hammett E., Chakraborty S., Quiles-Torres C., Selim M.A., Leong K.W., Levinson H. (2016). Biostable electrospun microfibrous scaffolds mitigate hypertrophic scar contraction in an immune-competent murine model. Acta Biomater..

[17] Sheikholeslam M., Wright M.E.E., Jeschke M.G., Amini-Nik S.J.A.H.M. (2018). Biomaterials for Skin Substitutes. Adv. Healthc. Mater..

[18] Chan J.P., Battiston K.G., Santerre J.P. (2019). Synthesis and characterization of electrospun nanofibrous tissue engineering scaffolds generated from in situ polymerization of ionomeric polyurethane composites. Acta Biomater..

[19] Guo F.Y., Wang N., Wang L., Hou L.L., Ma L., Liu J., Chen Y., Fan B.B., Zhao Y. (2015). An electrospun strong PCL/PU composite vascular graft with mechanical anisotropy and cyclic stability. J. Mater. Chem. A.

[20] Vilay V., Mariatti M., Ahmad Z., Pasomsouk K., Todo M. (2009). Characterization of the mechanical and thermal properties and morphological behavior of biodegradable poly(L-lactide)/poly(ε-caprolactone) and poly(L-lactide)/poly(butylene succinate-co-L-lactate) polymeric blends. J. Appl. Polym. Sci..

[21] Kim M., Hong B., Lee J., Kim S.E., Kang S.S., Kim Y.H., Tae G. (2012). Composite System of PLCL Scaffold and Heparin-Based Hydrogel for Regeneration of Partial-Thickness Cartilage Defects. Biomacromolecules.

[22] Jeong S.I., Kwon J.H., Lim J.I., Cho S.-W., Jung Y., Sung W.J., Kim S.H., Kim Y.H., Lee Y.M., Kim B.-S. (2005). Mechano-active tissue engineering of vascular smooth muscle using pulsatile perfusion bioreactors and elastic PLCL scaffolds. Biomaterials.

[23] Zhao X.J. (2016). Preparation of Carboxymethyl Chitosan Based Composite Materials and Their Application in the Repair of Bone Defect. Ph.D. Thesis.

[24] Gao X., Wen M.L., Liu Y., Hou T., An M.W. (2022). Mechanical performance and cyocompatibility of PU/PLCL nanofibrous electrospun scaffolds for skin regeneration. Eng. Regen..

[25] Alavarse A.C., de Oliveira Silva F.W., Colque J.T., da Silva V.M., Prieto T., Venancio E.C., Bonvent J.J. (2017). Tetracycline hydrochloride-loaded electrospun nanofibers mats based on PVA and chitosan for wound dressing. Mater. Sci. Eng. C Mater. Biol Appl.

[26] Adeli H., Khorasani M.T., Parvazinia M. (2019). Wound dressing based on electrospun PVA/chitosan/starch nanofibrous mats: Fabrication, antibacterial and cytocompatibility evaluation and in vitro healing assay. Int. J. Biol. Macromol..

[27] Wen M.L., Zhi D.K., Wang L.N., Cui C., Huang Z.Q., Zhao Y.H., Wang K., Kong D.L., Yuan X.Y. (2020). Local Delivery of Dual MicroRNAs in Trilayered Electrospun Grafts for Vascular Regeneration. Acs. Appl. Mater. Interfaces.

[28] Rafique M., Wei T., Sun Q., Midgley A.C., Huang Z., Wang T., Shafiq M., Zhi D., Si J., Yan H. (2021). The effect of hypoxia-mimicking responses on improving the regeneration of artificial vascular grafts. Biomaterials.

[29] Ababzadeh S., Farzin A., Goodarzi A., Karimi R., Sagharjoghi Farahani M., Eslami Farsani M., Gharibzad K., Zahiri M., Ai J. (2020). High porous electrospun poly(ε-caprolactone)/gelatin/MgO scaffolds preseeded with endometrial stem cells promote tissue regeneration in full-thickness skin wounds: An in vivo study. J. Biomed. Mater. Res. B.

[30] Zhong J.C., Zhang Y.J., Chen J.F., Huang R.Y., Yang Y.K., Chen H.X., Huang Y., Tan W.H., Tan Z.K. (2018). In Vitro Study of Colon Cancer Cell Migration Using E-Jet 3D Printed Cell Culture Platforms. Macromol. Biosci..

[31] Deng L.L. (2019). Fabrication of Electrospun Gelatin Composite Nanofibers and Its Relevant Application. Ph.D. Thesis.

[32] Yan H.Y., Mi X.Y., Midgley A.C., Du X.C., Huang Z.Q., Wei T.T., Liu R.H., Ma T.Z., Zhi D.K., Zhu D.S. (2020). Targeted Repair of Vascular Injury by Adipose-Derived Stem Cells Modified with P-Selectin Binding Peptide. Adv. Sci..

[33] Zhou F., Wen M.L., Zhou P.Q., Zhao Y.H., Jia X.L., Fan Y.B., Yuan X.Y. (2018). Electrospun membranes of PELCL/PCL-REDV loading with miRNA-126 for enhancement of vascular endothelial cell adhesion and proliferation. Mat. Sci. Eng. C-Mater..

[34] Zhou Y.Z., Shen Q.C., Lin Y., Xu S.Y., Meng Q. (2020). Evaluation of the potential of chimeric spidroins/poly(L-lactic-co-ε-caprolactone) (PLCL) nanofibrous scaffolds for tissue engineering. Mat. Sci. Eng. C-Mater..

[35] Tan C.K. (2020). Research on the Preparation and Characterization of CS/PVA-alginate Composite Hemostatic Dressing. Master Thesis.

[36] Li D.Y. (2017). A Study on the Wet Forming Craft of Chitosan/Polyvinyl Alcohol Blending Film Based on DOE and Its Crosslinking Modification. Master’s Thesis.

[37] Yang L.L., Pijuan-Galito S., Rho H.S., Vasilevich A.S., Eren A.D., Ge L., Habibović P., Alexander M.R., de Boer J., Carlier A. (2021). High-Throughput Methods in the Discovery and Study of Biomaterials and Materiobiology. Chem Rev.

[38] Wang Z.W. (2018). Cell Response and Mechanical Properties of the Nanofibers Reinforced Bilayered Tissue Guided Memberanes. Master’s Thesis.

[39] Luebberding S., Krueger N., Kerscher M. (2013). Mechanical properties of human skin in vivo: A comparative evaluation in 300 men and women. Ski. Res. Technol..

[40] Morgado P.I., Aguiar-Ricardo A., Correia I.J. (2015). Asymmetric membranes as ideal wound dressings: An overview on production methods, structure, properties and performance relationship. J. Membr. Sci..

[41] Wang X.M., Cheng F., Gao J., Wang L. (2020). Effect of cross-linking modification on properties of chitosan/polyoxyethylene nanofiber membranes towards wound care. J. Text. Res..

