# Supplementary material for: Synthesis and Characterization of PU/PLCL/CMCS Electrospun Scaffolds for Skin Tissue Engineering
Source: Polymers (Basel). 2022 Nov 19;14(22):5029. doi: 10.3390/polym14225029 (PMC9699124; doi:10.3390/polym14225029)
Supplement: Supplementary file 1 [file polymers-14-05029-s001.zip › polymers-2021572-supplementary.pdf]

Supporting Information

Synthesis and characterization of PU/PLCL/CMCS electrospun scaffolds for skin tissue engineering

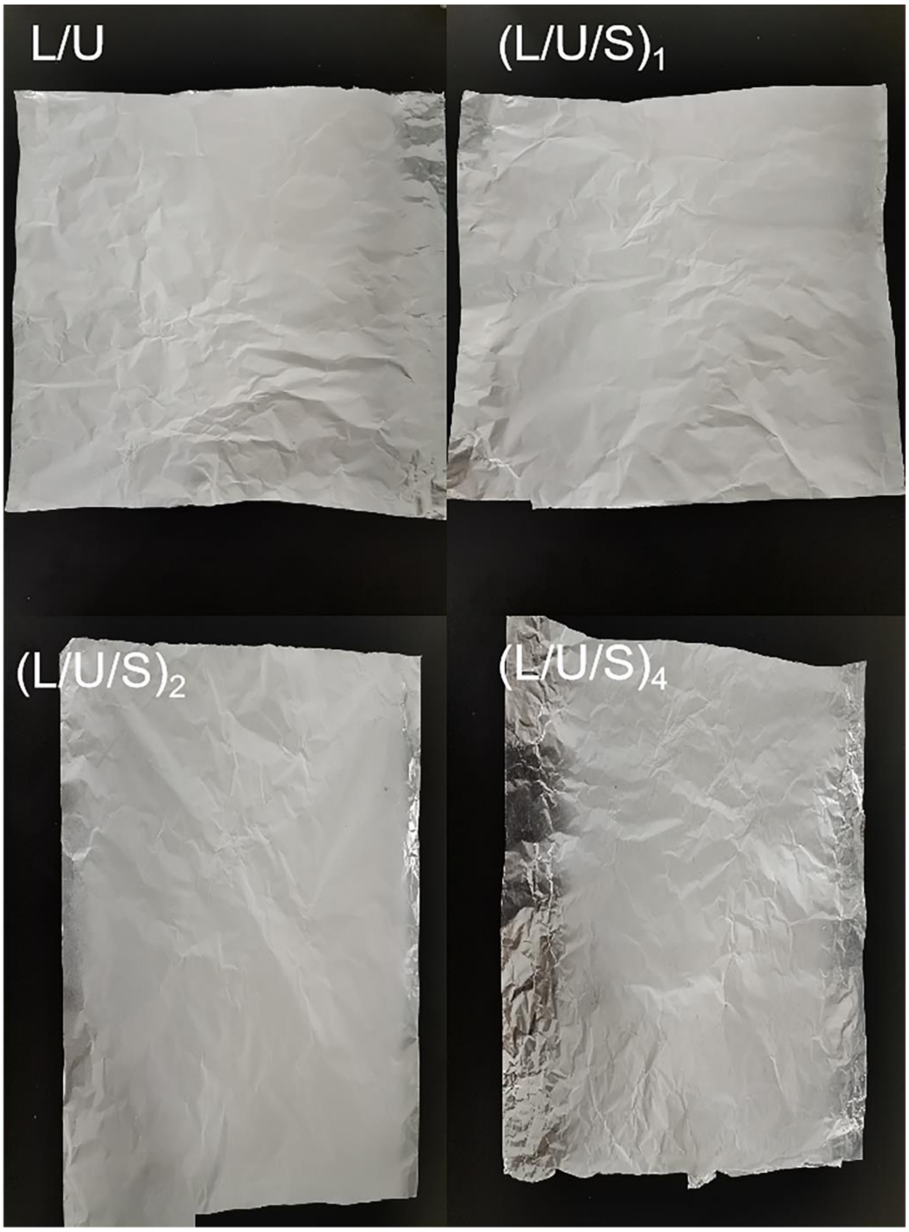

Figure S1. Visual images of the electrospun fiber membrane prepared with different CMCS content.

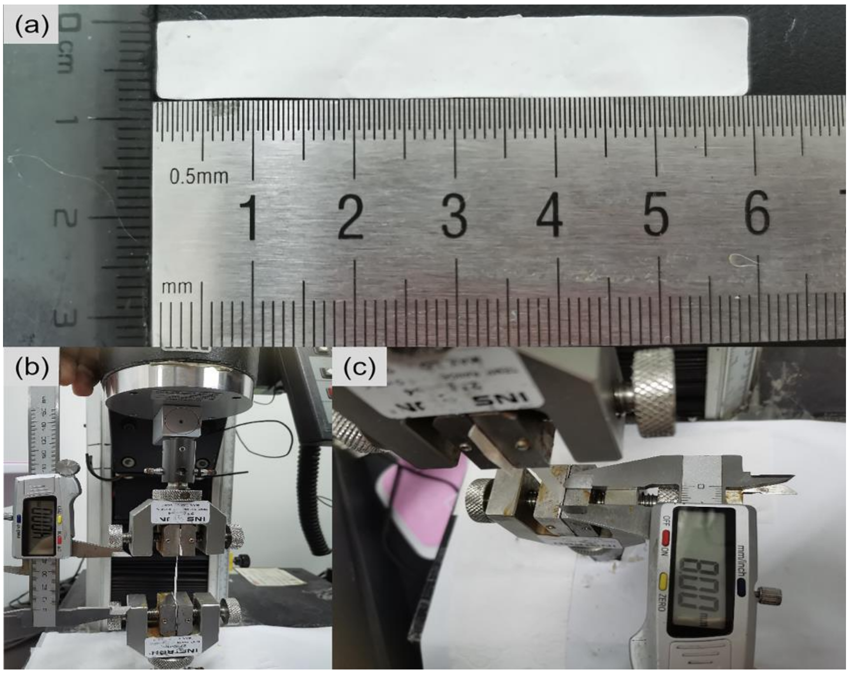

Figure S2. Original dimension of the electrospun membrane sample before stretching.

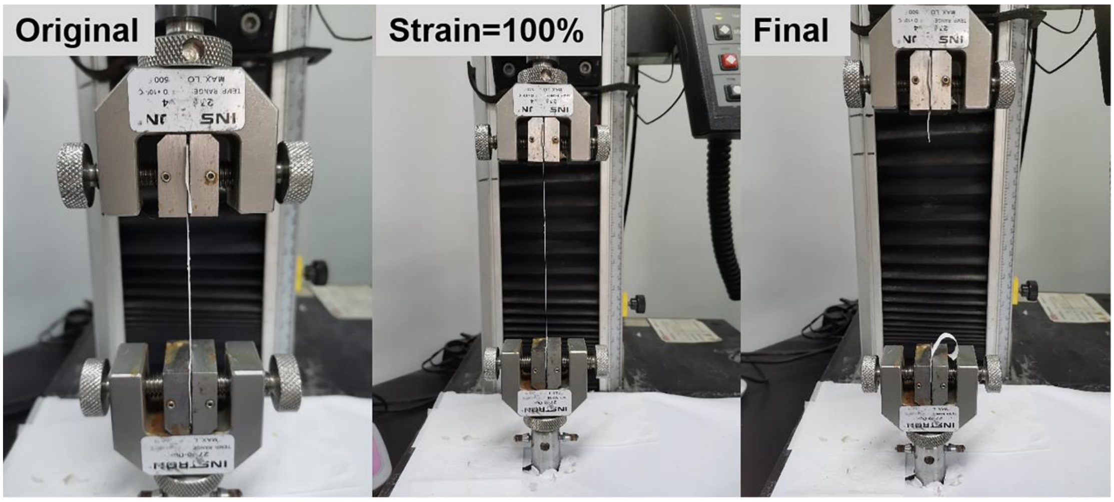

Figure S3. Images of the electrospun membrane sample in the stretching process.

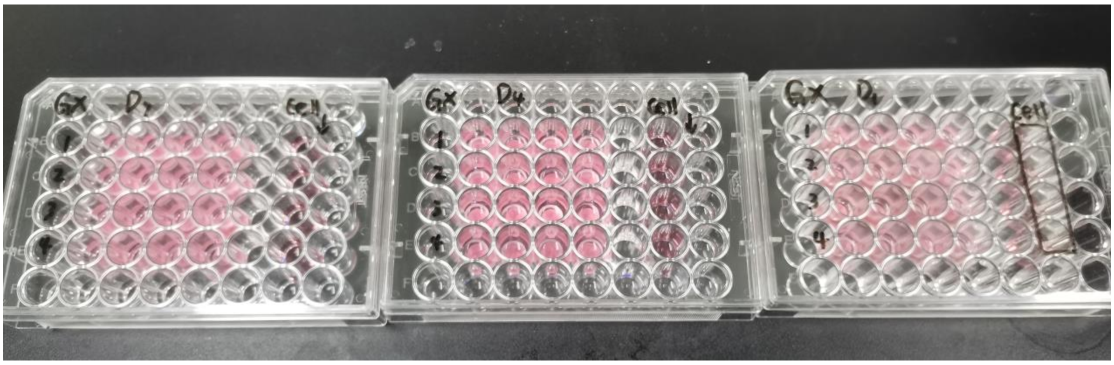

Figure S4. Image of cell culture plated in the methods CCK-8.
